# Allele Frequencies of Variants in Ultra Conserved Elements Identify Selective Pressure on Transcription Factor Binding

**DOI:** 10.1371/journal.pone.0110692

**Published:** 2014-11-04

**Authors:** Toomas Silla, Katrin Kepp, E. Shyong Tai, Liang Goh, Sonia Davila, Tina Catela Ivkovic, George A. Calin, P. Mathijs Voorhoeve

**Affiliations:** 1 Cancer and Stem Cell Biology Program, Duke-NUS Graduate Medical School, Singapore, Singapore; 2 Human Genetics, Genome Institute of Singapore, Singapore, Singapore; 3 Department of Medicine, National University of Singapore, Singapore, Singapore; 4 Cardiovascular & Metabolic Disorders Program, Duke-NUS Graduate Medical School, Singapore, Singapore; 5 Experimental Therapeutics & Cancer Genetics, MD Anderson Cancer Center, Texas State University, Houston, Texas, United States of America; 6 Division of Molecular Medicine, Ruder Boskovic Institute, Zagreb, Croatia; Inserm U869, France

## Abstract

Ultra-conserved genes or elements (UCGs/UCEs) in the human genome are extreme examples of conservation. We characterized natural variations in 2884 UCEs and UCGs in two distinct populations; Singaporean Chinese (n = 280) and Italian (n = 501) by using a pooled sample, targeted capture, sequencing approach. We identify, with high confidence, in these regions the abundance of rare SNVs (MAF<0.5%) of which 75% is not present in dbSNP137. UCEs association studies for complex human traits can use this information to model expected background variation and thus necessary power for association studies. By combining our data with 1000 Genome Project data, we show in three independent datasets that prevalent UCE variants (MAF>5%) are more often found in relatively less-conserved nucleotides within UCEs, compared to rare variants. Moreover, prevalent variants are less likely to overlap transcription factor binding site. Using SNPfold we found no significant influence of RNA secondary structure on UCE conservation. All together, these results suggest UCEs are not under selective pressure as a stretch of DNA but are under differential evolutionary pressure on the single nucleotide level.

## Introduction

Evolutionary conservation has been a good measure to identify potentially important regions in DNA and protein primary sequences [1]. In general, protein-coding genes, especially exons, are well conserved across species. Interestingly, a substantial fraction of non-coding regions are also strongly conserved. In fact, it has been proposed that conserved non-coding regions comprise approximately 1–2% of the human genome, about the same size as the coding regions [2]. Ultra Conserved Genes or Elements (UCGs, UCEs) are extreme examples among these conserved non-coding regions. In 2004 Bejerano et. al defined 481 UCEs in the human genome as regions 100% conserved over at least 200 base pairs when comparing human, mouse and rat reference genomes [3]. These UCEs comprise a subset of extremely conserved DNA elements; regions defined by extremely low nucleotide substitution rates across species [3], [4]. Although different publications come to different numbers of UCEs, it is clear that, depending on which genomes are compared and which conservation stringency filters are used, there are at least several thousand extremely constrained elements or genes present in the human genome [4]–[7]. Along with different conservation scores and filtering strategies, different groups have used many alternative names for these constrained elements. In this study we will collectively denote them as Ultra Conserved Elements or UCEs.

The extreme conservation of UCEs reflects a strong negative selective pressure on these regions [8], and by inference they are thought to have a critical function in metazoans, although the nature of this function is still enigmatic [9]. UCEs co-localize significantly with genes of specific ontological classes suggesting they may have gene regulatory functions [3], [4]. Analysis of fish genomes which underwent a genome duplication event shows that UCEs retain their conservation *in cis* in clusters spanning hundreds of kb's [6], often encompassing orthologous genes. This supports a model in which UCEs can act in concert as long range enhancers [4], [10], [11], and would explain their juxtaposition to developmental regulators. Further supporting the enhancer function of UCEs are the observations that UCEs can act as developmental enhancers and ‘hubs’ for transcription factor binding sites [4], [9], [10], [12]. However, the reason for their extreme conservation is not evident from this enhancer activity, especially since other enhancers have been described which are functionally strongly conserved without a concomitant conservation in their primary sequence [9].

Indeed, a different line of evidence suggests that UCEs produce functional non-coding RNAs [13]–[17]. UCEs were found to differentially generate long (>200 nt) RNA transcripts during development or in cancer [13], [15], [16]. Some of these RNA components seem functional, as siRNAs against RNAs transcribed from highly conserved elements that act as p53-dependent long range enhancers diminished enhancer activity [18]. Moreover, siRNAs against a UCE-derived RNA overexpressed in a cancer cell line induced apoptosis [13]. However, the molecular function of ultra-conserved RNA products is unknown.

UCEs have been shown to be under negative selection in the human population, since they are relatively devoid of common SNPs, and are depleted among segmental duplications and copy number variations [8], [19]–[21]. However, the level of negative selection has been debated. Some studies observed selection coefficients comparable to those of protein-coding regions or even stronger [8], [19]. A study, based on early genome data which found 24 SNPs in the 481 originally defined UCGs, failed to find strong, ongoing selection within UCEs and argued that the average level of selection on UCEs is less than that on essential genes [20]. Alternatively, this could suggest that some positions in UCEs are less constrained than others, and that UCEs are not under selective pressure as a continuous stretch of DNA. Neutral positions in an UCE could be due to relaxed evolutionary constraint in this particular UCE during recent human development, or in other words if the function of a UCE has recently become irrelevant for survival in modern humans, any variant would be neutral. On the other hand, neutral positions within UCEs could be due to different contribution of individual nucleotides to the structure-function relationship within a UCE. These positions would be expected to be less severely conserved if their identity contributes indeed less to UCE function. Regardless of the basis of the relaxed constraint in the human population, variations in less detrimental positions have a higher chance to become prevalent in human populations. To reliably address these questions, unbiased information on the occurrence and prevalence of variations in UCEs from different populations is required. Moreover, identifying the majority of prevalent SNVs [minor allele frequency (MAF)>5%] and accurately defining rare variants (MAF<0.5%), requires hundreds of samples from each population. We characterized natural variations in 2884 UCEs in two distinct populations comprised of 280 Singaporean Chinese and 501 Italians using a pooled capture sequencing approach. We observed that UCEs indeed contain positions that have potentially lower impact on UCEs function, suggesting differential evolutionary pressure on the single nucleotide level.

## Materials and Methods

### Samples

We used 280 DNA samples from ethnic Chinese (n = 141 males, n = 139 females) from the Singapore Prospective Study Program (SP2) [22]. 501 Italian samples consisted of 126 DNA samples from Italian female blood donors recruited through the Immunohematology and Transfusion Medicine Service of INT Milan and 375 female patients affected with invasive breast cancer collected through the Medical Genetics Unit of the INT Milan [23]. Based on the grounds that pooling strategy anonimizes each sample, exemption from IRB approval was obtained from the National University of Singapore Institutional Review Board (NUS-IRB, reference code 10-298E). The same approval waived the need for written informed consent from the participants.

### UCEs selection

Our UCEs selection combined the regions from three different studies- [3]–[5] using “merge genomic intervals” in Galaxy [24]. Final selection of studied UCEs is shown Table S1.

### Pooling and sample preparation

From the Singapore Chinese samples we created 18 pools. Each pool contained 14–16 samples. From the Italian female samples we created 48 total pools, 10–11 samples per pool. The DNA concentration of each individual sample was determined by qPCR (Singapore Chinese samples) or by NanoDrop instrument (Italian samples). Samples were added in equal molar concentrations of DNA in pools to obtain a final pool DNA amount of 1 µg per pool. Pools were then carried through the standard Illumina library preparation process. Briefly, the genomic DNA in the pools was sheared using Covaris S2, followed by end repair, A-tailing and ligation of unique Illumina indexes for each pool (New England Biolabs enzymes were used).

### Capture and next generation sequencing

Prepared libraries were combined in equal amounts (as determined by qPCR with adapter primers) and captured using Roche NimbleGen custom designed SeqCap EZ Library solution-based capture reactions and the recommended protocol. The total captured genomic region was ∼1.39 Mb. Table S1 lists all the regions that we captured and studied. After capture and PCR enrichment, pools were sequenced on the Illumina HiSeq2000 platform using a 101 bp paired end (PE) multiplexed read protocol.

### Mapping and variant calling

Reads from the pools were aligned to the UCSC human reference genome (hg19) using BWA aligner (version 0.5.6) with default parameters [25]. PCR duplicates were removed using MarkDuplicates in the Picard package (http://picard.sourceforge.net). Thereafter base quality score recalibration and realignment of the reads around the indels was performed using tools BaseRecalibrator and IndelRealigner form the GATK package [26], [27]. The list of 1000 G phase 1 indels and dbSNP135 variants were used to guide the realignments and base quality score recalibration, respectively. Indel and ddbSNP135 guide files were downloaded form the GATK bundle Variant calling was performed with programs CRISP [28] and UnifiedGenotyper (GATK package). CRISP is specifically designed for SNVs and indel discovery from pooled sequencing datasets and has shown to outperform some other widely used methods [28]. The GATK UnifiedGenotyper is a well -recognized and -supported SNVs and indel caller that was initially intended for variant calling from single sample next-generation sequencing datasets that now has an additional option for calling variants from pooled datasets. UnifiedGenotyper called variants were filtered by VariantFiltration (GATK package) using following filter expressions: "MQ <40.0", "FS>60.0", "MQRankSum <−12.5", "ReadPosRankSum <−8.0", "mpql", "StBias", "MQRS", "RdPS". For the final analysis we used only SNVs that were called by both programs (Figure S1). We used allele frequencies predicted by CRISP to discriminate prevalent (MAF>5%) and rare (MAF<0.5%) SNVs.

### phyloP score extraction

The 46-way placental alignment phyloP conservation scores were retrieved from the UCSC Genome archive (http://hgdownload.cse.ucsc.edu/goldenPath/hg19/phyloP46way/) through UCSC public MySQL server using hgWiggle.

### TFBSs overlap

TFBSs ENCODE track "wgEncodeRegTfbsClusteredV2" that combines TFBSs data across the cell types was downloaded from the UCSC server ftp://hgdownload.cse.ucsc.edu/goldenPath/hg19/encodeDCC/wgEncodeRegTfbsClustered/and used for the overlap studies [29], [30]. All Italian, Singapore-Chinese and 1000 Genome Project's rare and prevalent variants were tested for overlap with the TFBSs and compared to randomly selected UCE positions (G/C content corrected). Random UCE sets contained equal number of positions to the rare or prevalent variants in the corresponding data sets.

For the Super-Enhancer SNVs overlap, one hundred sets of 1000 random rare or prevalent SNVs were selected and each set was tested for TFBSs overlap. From this the mean overlap and 95% confidence intervals was calculated. Random positions selection and all overlap studies were done by BEDTools shuffleBed and intersectBed tools, respectively [31].

### External datasets

1000 Genome phase1 version3 variants were downloaded from ftp://ftp.1000genomes.ebi.ac.uk/vol1/ftp/release/20110521/ [32].

Super-enhancer regions were obtained from the study by Whyte et.al [33]. Mouse to human conversion was done by the liftOver tool (http://genome.ucsc.edu/cgi-bin/hgLiftOver).

### RNA secondary structure prediction

RNA secondary structure predictions were made by RNAsnp perl script which allows large-scale analysis [34]. RNSsnp Mode 1 was used with default parameters, except minimum length of the sequence interval was set to 25. Parameter descriptions can be found from the RNAsnp Web Server (http://rth.dk/resources/rnasnp/).

### Statistical analyses

All statistical calculations and figures were produced using R and its packages.

## Results

### General characterization of single nucleotide variations within UCEs from three different data sets

We analyzed single nucleotide variations (SNVs) within 2884 UCE regions that span ∼1.39 Mb of the human genome (Table S1). In order to resequence this relatively large set of elements in a comprehensive and economical manner we used a pooled sample targeted sequencing strategy, a cost effective and reliable approach for population resequencing studies [35], [36]. We had access to samples from two different ancestries: 501 samples from an Italian population (ITA) and 280 samples from a Singaporean Chinese cohort (SG-CHN) (Materials and Methods). All samples were pooled by population and gender with each pool containing 14–16 samples from the Singapore Chinese cohort or 10–11 samples from the Italian cohort. Pooled samples were then carried through a library preparation protocol as though they were from a single genomic sample. For the enrichment reactions, pools from the same population were combined. After enrichment, all pools were sequenced on the Illumina HiSeq2000 platform using a 101 bp PE multiplexed read protocol. On average, each ITA pool had 430- fold coverage and each SG-CHN pool 848- fold coverage across the targeted regions. This translates to an average 20-fold and 27-fold coverage per each sequenced allele, respectively. More then 97% of sequenced alleles were covered five or more times (Table S2). This sequencing depth has been shown to be sufficient to discover more than 90% of variants with low false discovery rate [28].

For variant calling, we used two independent variant calling programs, namely CRISP [28] and UnifiedGenotyper from the GATK package [26]. Both methods have been used by several studies for detecting variations from pooled samples [36], [37]. In this study we focused on SNVs. To obtain a confident list of SNVs for further analysis, we used the overlap of SNVs that were called by both algorithms. As expected, we noticed that the vast majority (96–97%) of all called SNVs were discovered by both methods regardless of sequenced population (Figure S1).

**Figure 1:**
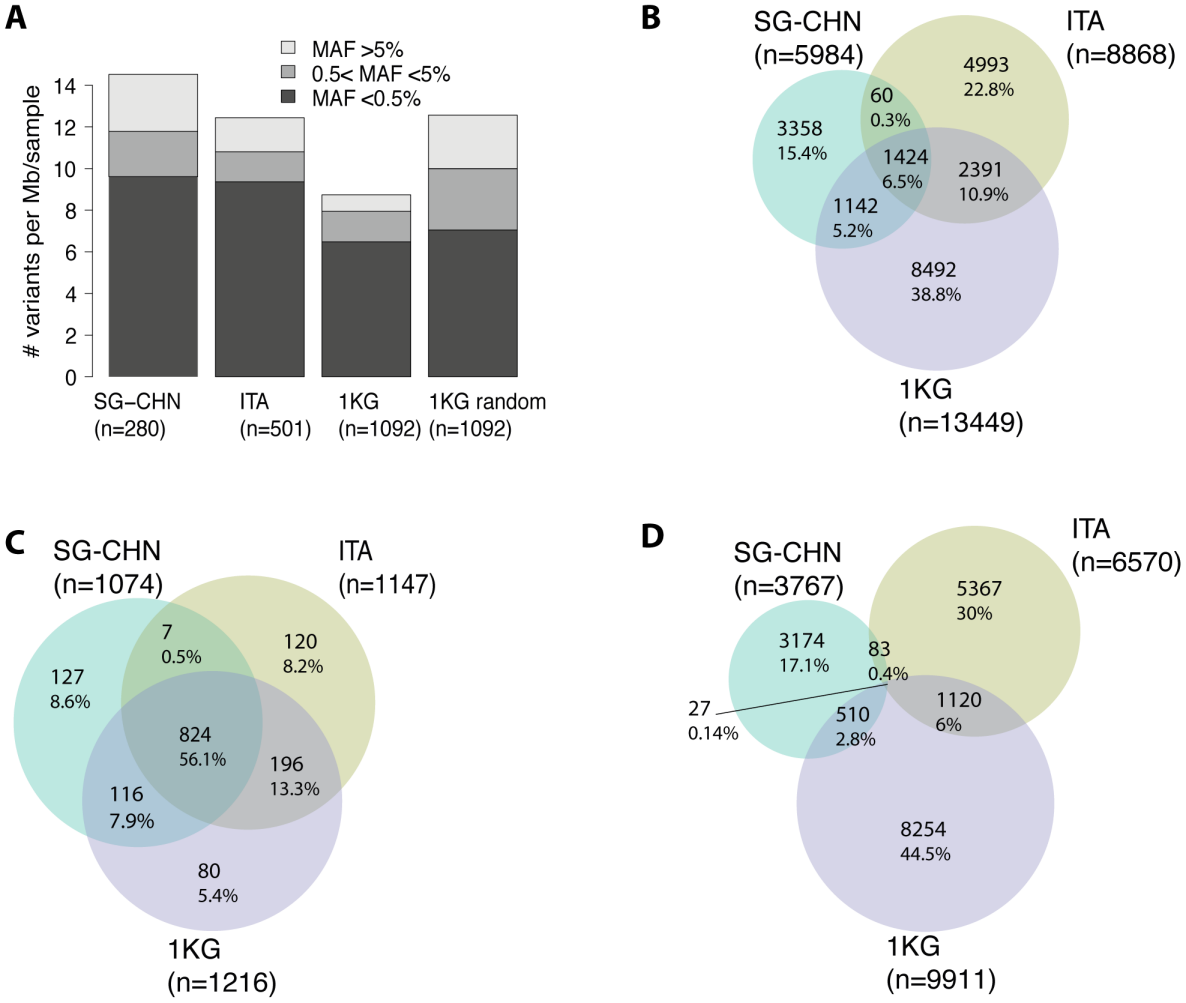
General characterization of SNVs in the UCEs. (**A**) Number of SNVs per mega base (Mb) of UCE sequence per sample. SNVs from three data sources- Singaporean Chinese cohort (SG-CHN), Italian cohort (ITA) and 1000 Genome Project (1 KG) were used. SNVs are discriminated according to their minor allele frequency (MAF). Numbers in the parentheses represent sample size used in this study (Materials and Methods). Random set represents random genomic regions that have the same total length as the UCEs set. Y-axis represents SNVs per Mb divided by sample count in the analyzed population. (**B–D**) Shared and distinct SNVs between SG-CHN, ITA and 1 KG populations. Venn diagrams of (**B**) all, (**C**) prevalent (MAF>0.5%) and rare (**D**) (MAF<0.5%) SNVs from three analyzed population. Numbers in the parentheses indicate analyzed SNVs in the corresponding population.

Within the targeted regions from the SG-CHN and ITA cohorts, we detected about 15.1 and 12.4 SNVs per one Mb of targeted region per sample, respectively (Figure 1A). In order to get an additional and independent data set for our study we also extracted all SNVs from the 1000 Genome project (1 KG) phase 1 variants within the defined UCE regions. This identified 13449 SNVs within the targeted regions from 1092 samples, which translates into 8.7 variants per one Mb of targeted region per sample (Figure 1A). From these numbers, it is evident that sequencing of more samples does not lead to a proportional increase of SNVs per sample. This is most likely due to detection saturation of prevalent variants and a relatively low number of private SNVs per sample (Figure 1A). Next, we discriminated SNVs according to their minor allele frequency (MAF) in the respective population. In concordance with previous results [8], [19], analysis of random genomic positions in the 1 KG dataset revealed depletion of prevalent SNVs (MAF>5%) in UCEs (Figure 1A, compare 1 KG to 1 KG random). Comparison of three datasets revealed that almost 1400 (6.5%) of all SNVs are present in all datasets (Figure 1B). A recent study that analyzed 202 protein coding genes in 14002 people revealed an abundance of rare SNVs compared to common variants [38]. Similarly, our study shows that majority of the detected SNVs in conserved non-coding regions are rare variants (MAF<0.5%) (Figure 1A). As expected, the majority (56%) of prevalent SNVs are present in all three datasets (Figure 1C). In contrast, only 27 rare SNVs (0.4%) are shared in all populations (Figure 1D).

### Prevalent and rare variants show distinctive conservation preference and potential functional consequences

**Figure 2:**
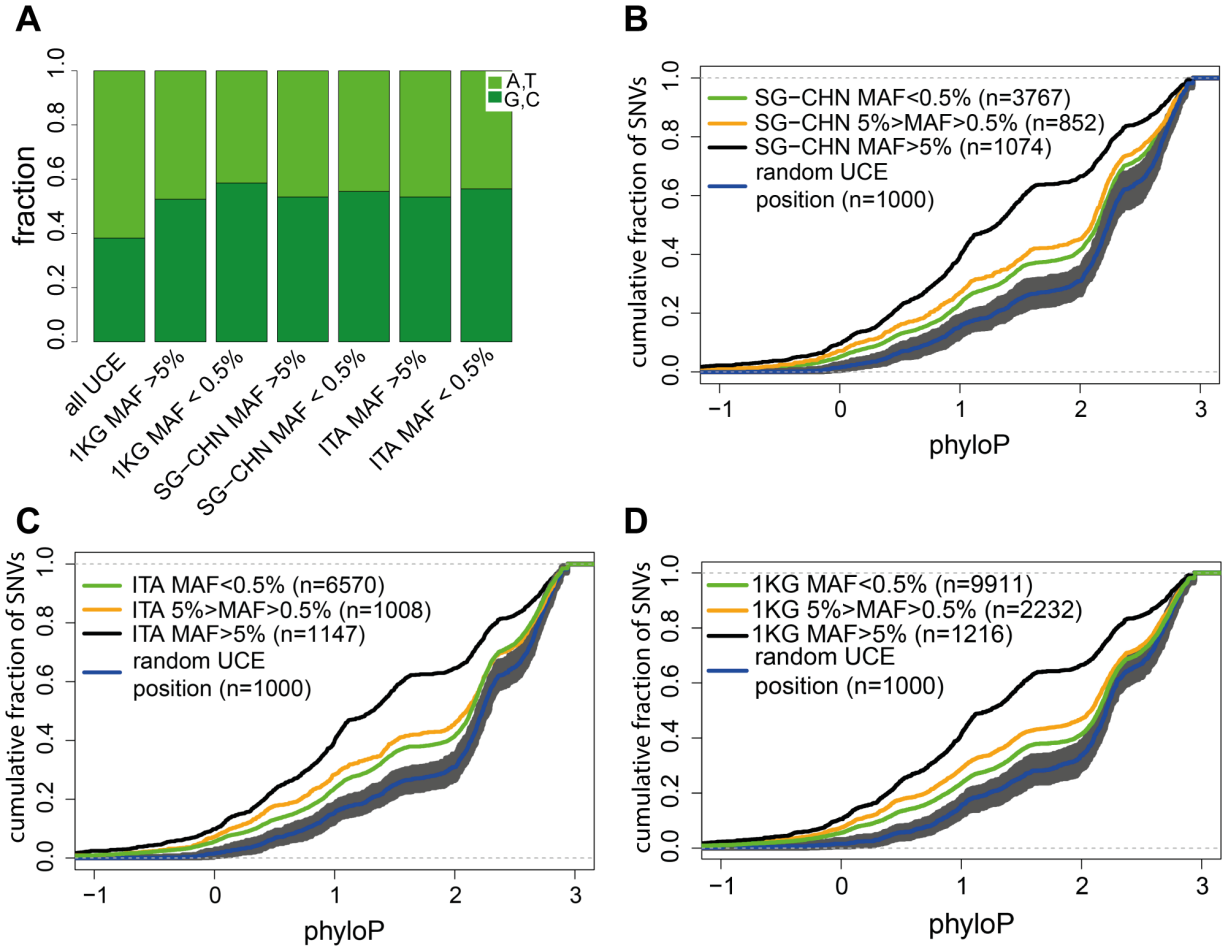
Prevalent and rare variants show distinctive conservation preference. (**A**) Distribution of A,T and G,C nucleotides in the UCEs and SNV positions. **(B–D**) Cumulative distribution plots of phyloP scores of SNVs with different MAFs. Data from three different data sources (**B**) SG-CHN, (**C**) ITA and (**D**) 1 KG are shown. Shaded grey area represents 95% confidence interval (obtained by bootstrapping) of random G/C content corrected UCE positions (blue line). Numbers in the parentheses indicate analyzed positions or SNVs.

Initial sets of UCEs were discovered by using the program PhastCons that finds conserved elements in multiple genome alignments [39]. An alternative to PhastCons is the phyloP method [40]. The most important difference between these two methods is that phyloP captures both conservation and acceleration of DNA positions and operates independently at each site, whereas PhastCons also considers neighboring positions for its calculations. PhyloP is thus more suitable to estimate selective pressure on the single nucleotide level. We decided to take advantage of the phyloP method to assess whether the prevalence of SNVs in the human population reflected different selective pressures during evolution. Indeed, prevalent SNVs have a lower conservation score compared to rare SNVs (P<2.2e−16 two-sided Kolmogorov–Smirnov test, Figure 2B–D). Importantly, this is true in all three data sets. SNVs with a MAF between 0.5% to 5% were also skewed towards lower conservation scores than the rare SNVs, which occur with a distribution that is very close to random. Altogether our data indicates that conservation score negatively correlates with SNVs prevalence in the population (Figure 2B–D).

Boundaries of UCEs are defined as a decline in the PhastCons conservation score. We excluded the possibility that prevalent SNVs are clustered closer to UCE boundaries by plotting the distance of each variant from its host UCEs start position (Figure S2). This analysis showed that both rare and prevalent SNVs are randomly distributed across the UCEs length (Figure S2), excluding border effects in the general lower phyloP score for prevalent SNVs.

Interestingly, rare SNVs also seem to occur in slightly less conserved positions compared to the random UCEs positions (Figure 2B–D). UCEs in general are known to be AT rich [41], however, SNVs are enriched in G or C positions (Figure 2A). Therefore, we compared rare SNVs to a G/C content matched random set (Figure 2B–D). We noticed that rare SNVs have moderate difference from the G/C matched random set, indicating that rare SNVs positions may not occur completely randomly or more sophisticated (dinucleotide etc.) corrections for the random sets are required. Nevertheless, prevalent SNVs that occur in>5% of the population show a clear pattern: they are relatively depleted in the most conserved nucleotides.

To determine whether the pattern of conservation of the rare and prevalent SNVs were unique to that position, or reflected a selective pressure on the local nucleotide composition, we determined the PhyloP score for the nucleotides immediately upstream or downstream of the identified SNVs. The neighboring nucleotides of prevalent SNVs are clearly more constrained (Figure S3). Taken together, these data indicates evolutionary pressure on the single nucleotide level.

**Figure 3:**
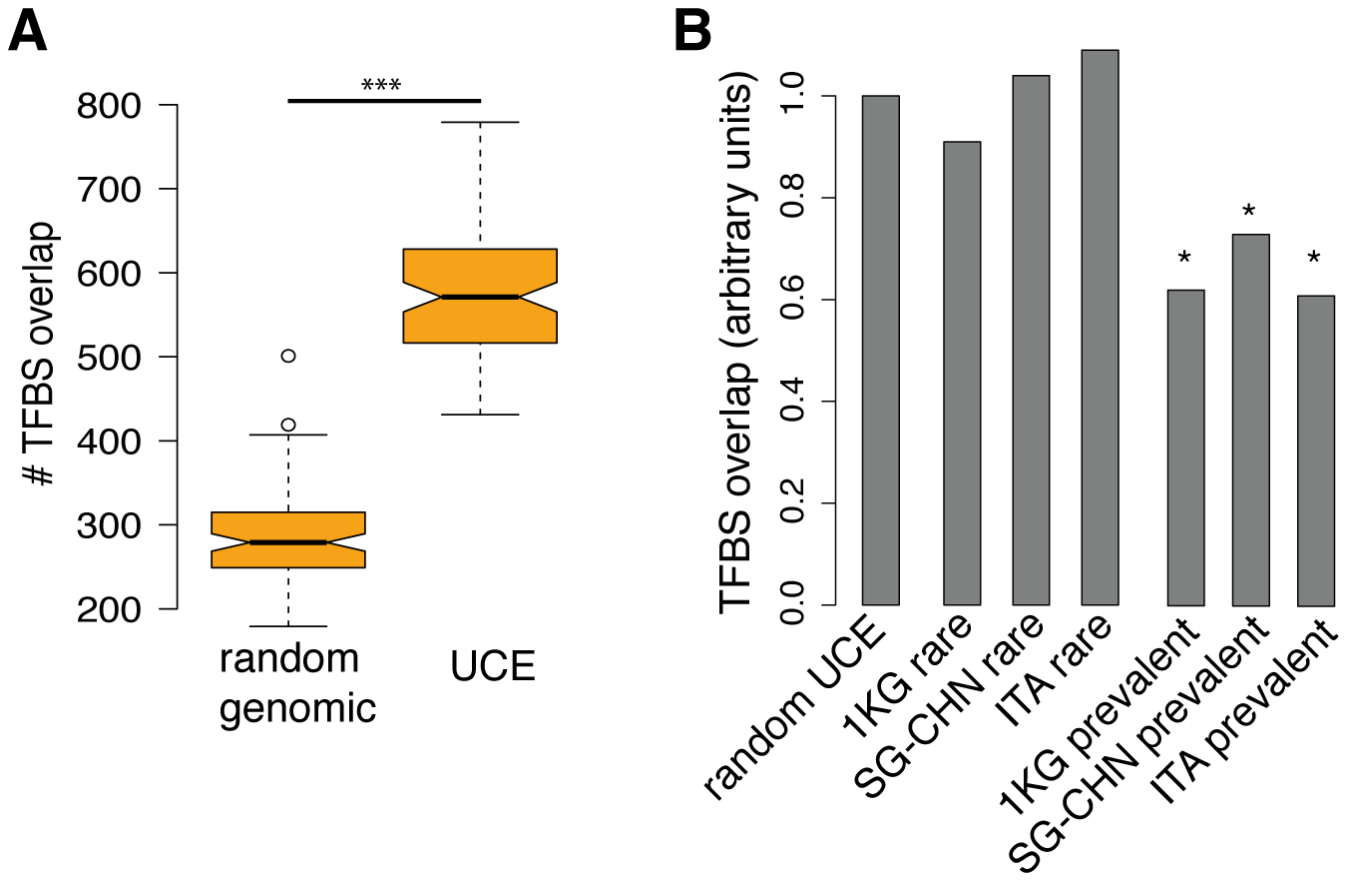
UCEs are enriched for the TFBS. (**A**) Box plots represent results of one hundred sets (each set contains one thousand randomly chosen positions). The y-axis indicates actual ENCODE TFBS overlap per one thousand tested positions. Boxes show IQR, notches indicate 95% confidence intervals of the median, whiskers extend to 1.5 times the IQR and open circles show outliers. *** P<2.2×10^−16^, two- tailed Mann–Whitney test. (**B**) Prevalent SNV positions are depleted for TFBS. All rare and prevalent SNV positions from the three different populations were analyzed for the ENCODE TFBS overlap. Random UCE set represents randomly chosen UCE positions (G,C content matched) that had the same number of analyzed positions as the rare and prevalent SNVs. Prevalent and rare SNVs overlap with the TFBS overlap is shown as relative to random UCE positions. For the statistical analysis each set (Pearson's Chi-squared test) was individually tested. * P<0.01.

Prevalent variations within UCEs may be tolerated because they have lower functional impact and hence these variations may spread in the population. UCEs are described to act as enhancers [4], [10], and therefore it is plausible that variations within UCEs may have impact on transcription factor (TF) binding. As part of an ENCODE consortium effort, a comprehensive set of human TF Binding Sites (TFBS) based on ChIP-seq experiments has been produced [42]. This dataset spans information from 91 human cell types and 161 unique regulatory factors. Consistent with the proposed role of UCEs, they are clearly enriched for TFBS from this dataset when compared with randomly shuffled genomic positions (Figure 3A). We decided to use this information in combination with the three above described independent datasets on SNV prevalence to test if we could detect a signature of selective pressure on TFBS in UCEs. Based on a possible functional selective pressure on enhancer activity, we speculated that prevalent variations present in UCEs are less likely to overlap with TFBS. Indeed, prevalent variations showed less overlap with TFBS than rare SNVs or random positions (G/C-matched) (Figure 3B, for all sets P<2.97e−10, Pearson's Chi-squared test). At the same time, the overlap of rare variants with TFBS is not significantly different from the random positions (Figure 3B). These results are consistent across the analyzed populations and indicate that TF binding may be one of the driving forces for the negative selection pressure on individual nucleotides within UCEs.

**Figure 4:**
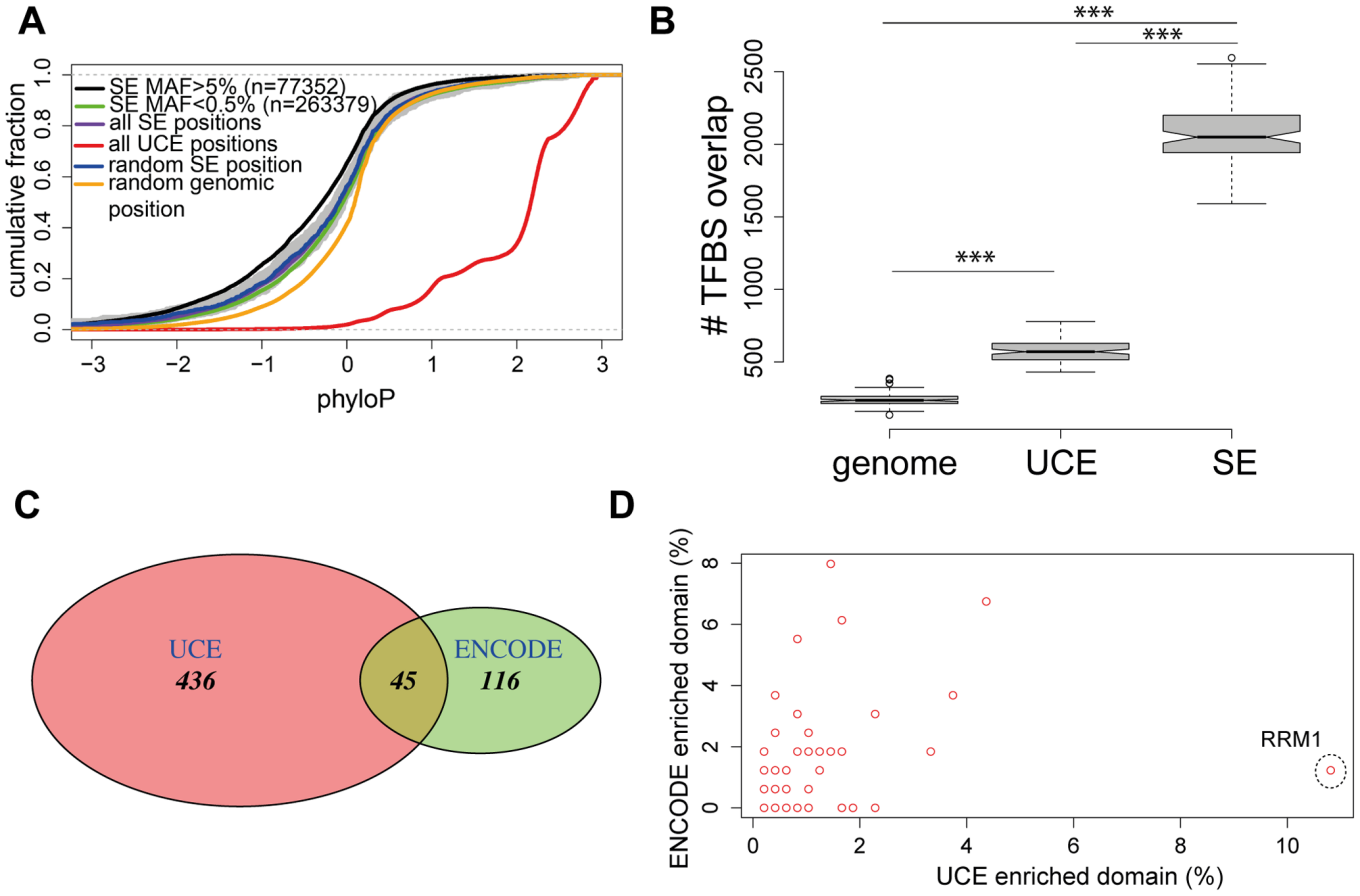
UCEs comparison to the less constraint SE. (**A**) SE are less constraint compared to UCEs. Cumulative distribution plots of phyloP scores of all SE positions (purple line), all UCE positions (red line), random genomic positions (orange line) and SE rare (MAF<0.5%, green line) and prevalent (MAF>5%, black line) SNVs. Prevalent and rare SNVs are extracted from the 1 KG project using global MAFs. Shaded grey area represents 95% confidence interval (obtained by bootstrapping) of random UCE positions (blue line). The numbers of analyzed SNVs are given in the parentheses. (**B**) SE have a higher overlapping TFBSs count compared to UCEs. Box plots represent results of one hundred sets (each set contains one thousand randomly chosen positions). The y-axis indicates actual ENCODE TFBS overlap per one thousand tested positions. Boxes show IQR, notches indicate 95% confidence intervals of the median, whiskers extend to 1.5 times the IQR and open circles show outliers. *** P <2.2 ×10^−16^, two- tailed Mann–Whitney test. (**C**) Venn diagram showing overlap of ENCODE TF and UCE bound TF described by Viturawong et. al [12]. (**D**) Comparison of ENCODE TF and UCE bound TF [12] protein domains identifies RNA recognition domain,RRM1 (marked with dashed circle), as the most prevalent domain among UCE bound proteins. Protein domain (Pfam) annotations were done by using the Perseus module in the MaxQuant software suite.

Next, we were curious to see whether the conservation score of SNVs within the non-constraint regulatory elements follows the same pattern as we have noticed for the UCEs. Super-Enhancers (SE) are densely occupied by TF and the Mediator co-activator [33], [43]. Contrary to UCEs, an evolutionary conservation analysis indicates that SE are not more conserved than random genomic positions (Figure 4A, P<2.2e−16 two-sided Kolmogorov–Smirnov test). We also extracted all 1 KG SNVs that occur in the SE and sub-grouped them according to MAF. This resulted in 77352 prevalent and 263379 rare SNVs, and translates to 9.3 SNVs per Mb. Interestingly, this is not significantly different from the SNVs count (8.7) per Mb of ultra-conserved sequence (Figure 1A). Nevertheless, similarly to UCEs, conservation analysis revealed that prevalent variants in SE occur in less conserved positions compared to the rare variants (Figure 4A, P<2.2e−16 two-sided Kolmogorov–Smirnov test).

A recent study showed that UCE are proposed to act as transcription factor binding “hubs” that contain multiple overlapping TFBS and this may count for the UCEs conservation [12]. Interestingly, we noticed that per 1000 randomly chosen positions, SE have a higher overlapping TFBSs count compared to UCEs (Figure 4B). This may indicate that UCEs are less densely occupied with transcription factors than other regulatory regions such as SE, or that UCEs bind different sets of proteins than those that were studied by the ENCODE consortium. The latter possibility likely contributes to this difference, since only 45 UCE bound proteins identified by Viturawong et. al [12] were studied in the ENCODE consortium (Figure 4C). To further address this difference we did domain enrichment analysis for both, ENCODE TFs and 481 proteins identified by Viturawong et. al [12]. We found that ∼10% of UCEs bound proteins contain the RNA recognition domain RRM1, whereas the same domain is present only in 1.2% ENCODE TFs (Figure 4D). All together, these results suggest that UCEs may bind a unique set of proteins. However, more importantly, the extreme conservation of UCEs is not solely explained by dense TFBS overlap.

### Analysis of UCEs encoded ncRNAs secondary structure

As enhancers, UCEs can also be transcribed to produce ncRNA [13]–[17]. Many ncRNAs fulfill their function through secondary structures that are recognized by other factors. Therefore we considered the possibility that selective pressure on RNA secondary structure may contribute to UCE conservation. To begin to address this hypothesis we used a recently developed program, RNAsnp, which predicts SNVs effects on local RNA secondary structure [34]. However, analysis of prevalent and rare variants from all three data sets did not show any significant difference in the RNAsnp reported p-values (Figure S4). Since RNA secondary structure prediction is complex with a high noise to signal ratio, we tested whether discriminating variations based on their conservation would result in a better discrimination. We noticed very moderate effects, but only when we compared the least constraint rare SNVs to the most constraint rare SNVs (Figure S4B). Although we observe the same trend in all analyzed datasets, the effect is not significantly different from the most constraint SE and random genomic SNVs (Figure S4B). Therefore, at this stage we are not able to convincingly prove that there is additional evolutionary pressure on the UCEs encoded ncRNA secondary structure.

## Discussion

The most striking feature of UCEs is the presence of so many consecutive conserved nucleotides. We can speculate that different positions in the UCEs are under different levels of selection. Thus, some positions may be neutral and with no or little effect on fitness. Indeed, we noticed that prevalent SNVs in the UCEs have in general lower phyloP score (based on 46 placental mammals) compared to the rare SNVs. Importantly, the same trend was true regardless of the analyzed population (Figure 2). This inverse correlation between SNVs occurrence and genomic conservation is not unique to UCEs. It has also been noticed in mRNA processing regions [44], but was not studied before in the context of the extremely constrained non-coding regions. These results show clearly that SNVs within UCEs occur randomly and each position may experience different levels of evolutionary pressure.

Does the conservation reflect the functional importance of each particular nucleotide? First of all, we noticed that UCEs contain an abundance of rare SNVs, 63–74% of all called variants have MAF less then 0.5% (Figure 1). One explanation for the abundance of rare variants is the rapid human population growth and weak purifying selection [38]. However, a second reason comes from the natural selection theory, which follows the logic that variants that are negatively affecting evolutionary fitness of an organism should be under purifying selection and be found only in the small fraction of population. Thus, more constrained positions, i.e. rare SNVs, should have more impact on the UCEs function then the prevalent SNVs that occur in the less constrained positions. Indeed, our analysis of prevalent and rare variants showed clearly that prevalent SNVs avoid TFBS compared to rare SNVs (Figure 3). These results are strong indication that more constrained rare SNVs occur in the UCE positions that are more likely to be functionally deleterious.

Our findings that prevalent SNVs are depleted for TFBS (Figure 3) support a recent study by Viturawong *et. al* that shows overlapping TFBS within their tested UCEs are more stringently conserved [12]. The same study proposed that heavily overlapping TFBSs could count for the UCEs conservation [12], although this theory has been previously questioned by others [9]. Our study confirmed that UCEs are enriched for TFBSs compared to the same size of random genomic regions (Figure 3). Interestingly, our analysis of SE, a recently described class of non-conserved enhancers that are densely occupied by master regulators and Mediator [33], showed that these elements have much higher TF overlap per tested nucleotide compared to UCEs (Figure 4B). In this respect, we can not conclude that UCEs are more enriched for TFBSs compared to the SE, leaving open additional, unknown, mechanistic pressures to contribute to their striking conservation.

Our study is the first that has specifically characterized natural variations in UCEs at this scale. There is emerging evidence that the non-coding genome, including regulatory regions, contribute to complex traits [45]–[48]. We propose that UCEs are good candidates for targeted sequencing projects and association studies. Indeed, a recent paper showed that the evolutionary constrained SNP rs6983267, that has been consistently associated with an increased risk of colorectal cancer, is hosted by conserved regulatory element that encodes ncRNA, and when encompassing the rs6983267 SNP promotes tumor growth, metastasis, and chromosomal instability [49]. Our study provides data to model expected background variations that can be used for power calculations of potential association studies. Moreover, we propose that conservation phyloP score might be relevant to prioritize potential disease-causing SNVs for replication and functional studies.

Why are UCEs so ultra conserved? We show a clear selective pressure that correlates with phyloP score, indicating that the pressures that were present during evolution of vertebrates were still relevant, at least until very recently, in the human population, with regard to UCEs. Although there is a clear signal indicating the importance of TF binding in UCEs, the density of in vivo validated TFBS in UCEs or the selection against higher conserved sites in prevalent SNVs, do not explain their conservation. Although production of functional RNA from UCEs is an attractive model for additional selection pressure parallel or synergistic with pressure to bind TF, we could not confirm a role of secondary RNA structure in our data. Better RNA prediction algorithms and/or in vivo structure studies of nuclear noncoding, low abundant RNAs are required to thoroughly address this question.

## Supporting Information

Figure S1 **Comparison of SNVs called by CRISP and GATK.** SNVs from the **(A)** SG-CHN and **(B)** ITA populations were called by programs CRISP and GATK (Materials and Methods). The overlapping regions of the Venn diagram indicate SNVs that are called by both programs. SNVs that are called only by CRISP or GATK are indicated by the red and green colors, respectively.
(EPS)

Figure S2 **Prevalent and rare SNVs are randomly distributed across the UCE sequence.** Black dots represent the distance (y-axis) of each UCE nucleotide and its corresponding phyloP score (x-axis) as the percentage from the start point of the UCE. UCE start is defined as 0% and end as 100%. UCE flanks (same length as the UCE, shown as the grey dots) represent upstream (100–200%) and downstream (−100–0%) regions from the UCE end and start, respectively. Location of prevalent and rare SNVs and their corresponding phyloP scores from the **(A)** 1 KG, **(B)** SG-CHN and **(C)** ITA populations are shown as green and red dots, respectively. UCE start and end coordinates are shown in the Table S1.
(EPS)

Figure S3 **Neighboring positions of prevalent SNVs are more constraint.** Cumulative fraction of phyloP scores of prevalent (MAF>5%) and rare (MAF<0.5%) SNVs from the **(A)** ITA, **(B)** SG-CHN and **(C)** 1 KG populations are shown as black and green lines, respectively. Upstream and downstream regions are defined as the three bp windows from the corresponding SNVs. Brown and red lines represent the cumulative faction of phyloP scores of the prevalent SNVs upstream and downstream regions, respectively. Orange and purple lines represent the cumulative faction of phyloP scores of the rare SNVs upstream and downstream regions, respectively. Shaded grey area represents 95% confidence interval (obtained by bootstrapping) of random UCE positions (blue line).
(EPS)

Figure S4 **Prediction of SNVs effects on the local RNA secondary structure.** **(A)** Analysis of prevalent and rare variants from all three data sets. **(B)** Analysis of rare most conserved (phyloP score>2) and least conserved (phyoP score <2) SNVs. y-axis represents RNAsnp [34] reported p-values. Mean (open circles) and 95% confidence limits of mean (red bars) are shown.
(EPS)

Table S1 **Genomic coordinates (hg19) of analysed UCEs.** Excel sheet containing genomic coordinates of analysed UCEs, including information about overlap with UCSC Genes 5′UTR, 3′UTR and exons.
(XLSX)

Table S2 **General characteristics of sequenced sample pools.** This excel sheet contains following information for each sequenced pool: mapped reads on target, average sequencing depth, average depth per allele, % of alleles covered> = 5X.
(XLSX)
